# Mediastinoscopy-assisted esophagectomy for T2 middle and lower thoracic esophageal squamous cell carcinoma patients

**DOI:** 10.1186/s12957-018-1361-2

**Published:** 2018-03-16

**Authors:** Jun Wang, Ning Wei, Yimin Lu, Xiaoying Zhang, Nanqing Jiang

**Affiliations:** The First People’s Hospital of Changzhou, The Third Affiliated Hospital of Soochow University, 185 Juqian Road, Changzhou, Jiangsu Province 213003 China

**Keywords:** Esophageal carcinoma, Ivor Lewis, Mediastinoscopy-assisted esophagectomy

## Abstract

**Background:**

We aimed to compare mediastinoscopy-assisted esophagectomy (MAE) with the Ivor Lewis procedure in T2 middle and lower thoracic esophageal carcinoma patients in fields of perioperative complications and overall survival (OS).

**Methods:**

The clinical data of 112 T2 esophageal cancer patients who received MAE (*n* = 31) or Ivor Lewis procedure (*n* = 81) from January 2010 to December 2015 were retrospectively analyzed in propensity score analysis. Thirty-eight T2 esophageal cancer patients who underwent MAE (*n* = 19) and Ivor Lewis procedure (*n* = 19) were included in this study. The perioperative conditions and OS were analyzed.

**Results:**

The MAE group showed shorter operation time (143.2 ± 20.6 vs 176.8 ± 31.1 min, *P* = 0.001), less drainage in 24 h (119.2 ± 235.1 vs 626.3 ± 396.3 mL, *P* < 0.001), less retention time of thoracic tube (27.8 ± 24.0 vs 101.2 ± 54.6 h, *P* < 0.001), and less hemorrhage during operation (255.4 ± 159.8 vs 367.4 ± 150.9 mL, *P =* 0.059) compared with the Ivor Lewis group. Less dissected lymph nodes were detected in the MAE group (12.2 ± 5.4 vs 16.8 ± 5.8, *P* = 0.044) than in the Ivor Lewis group, especially in the upper mediastinum (1.8 ± 2.1 vs 3.5 ± 2.3, *P* < 0.001) and middle mediastinum (2.5 ± 2.0 vs 5.3 ± 3.2, *P* = 0.027). The mean survival time was 59.1 and 53.3 months for the MAE group and Ivor Lewis group, respectively (*P* = 0.635). The results of Cox regression indicated that the nodal stage (*P* = 0.016) was an independent prognostic factor and the surgical method was not an independent prognostic factor for these patients (*P* = 0.290).

**Conclusions:**

MAE procedure showed less surgical trauma compared with the Ivor Lewis procedure. The mediastinal lymphadenectomy of T2 esophageal carcinoma patients who underwent MAE was inferior to those who underwent Ivor Lewis procedure. The perioperative complications and OS of the MAE group were no worse than that of the Ivor Lewis group.

## Background

Esophageal carcinoma is a common malignancy with high morbidity and mortality worldwide [1–3]. Esophageal carcinoma was with about 0.48 million new cases and 0.44 million deaths in 2015 [4]. The morbidity of esophageal carcinoma is estimated to increase in the future [5]. China has the most esophageal carcinoma patients of the entire world [6]. The esophageal carcinoma is the sixth most common malignancy in China. And the mortality of esophageal carcinoma ranks fourth among all malignant tumors in China [6]. Esophageal carcinoma can be divided into two main subtypes: esophageal adenocarcinoma and esophageal squamous cell carcinoma (ESC). Most esophageal carcinoma patients in the western country suffered from esophageal adenocarcinoma [7]. The incidence of ESC was more than 90% in China [8]. The middle and lower thoracic esophageal carcinoma patients account for more than 80% of all esophageal carcinoma patients in China [7]. Esophagectomy is an important modality for ESC treatment. The controversies about the operative approach of esophagectomy still continue [9]. Both the Ivor Lewis esophagectomy (Ivor Lewis) and the mediastinoscopy-assisted esophagectomy (MAE) have been performed in our department for a long time. MAE was reported by Buess and Becker in 1990 [10]. It is still controversial because of the insufficient mediastinal lymphadenectomy in MAE procedure [11]. Our previous study showed that MAE was suitable for T1 esophageal carcinoma patients and had an optimal longtime survival rate [12]. On this basis, MAE has been performed for esophageal carcinoma patients with preoperative stage T1 in our department since 2010. The potential differences between the preoperative T stage and the postoperative T stage cause some unexpected T2 patients received MAE in our department [13]. This study aimed to compare MAE with Ivor Lewis procedures in T2 esophageal carcinoma in terms of perioperative complications, postoperative pathological findings, and long-term survival rate.

## Methods

From January 2010 to December 2015, T2 esophageal carcinoma patients confirmed by postoperative pathological examination, who underwent MAE or Ivor Lewis procedure in Changzhou First People’s Hospital, were enrolled in this study.

The operations were carried out by the experienced surgeons with having performed more than 80 MAE and more than 100 Ivor Lewis esophagectomies. Patients were included only if they satisfied the following criteria: (a) not suffered from other malignant diseases, (b) without history of gastric or esophageal surgery, (c) no major organ dysfunction, (d) Karnofsky Index score greater than or equal to 90, (e) no history of neoadjuvant chemotherapy or radiotherapy, (f) without distant metastasis, and (g) no history of endoscopic therapies. All patients underwent detailed history collection and physical examination pre-operation. The pulmonary function tests, arterial blood gas analysis, serum biochemical indexes, coagulation indexes, and cardiac ultrasonography were performed for all patients. The diagnoses were all identified by endoscopy and biopsies. CT scans of the cervical region, thorax, and upper abdomen were obtained for all patients. This study was approved by the Clinical Research Ethics Committee of our hospital.

### Surgical procedure

**Fig. 1:**
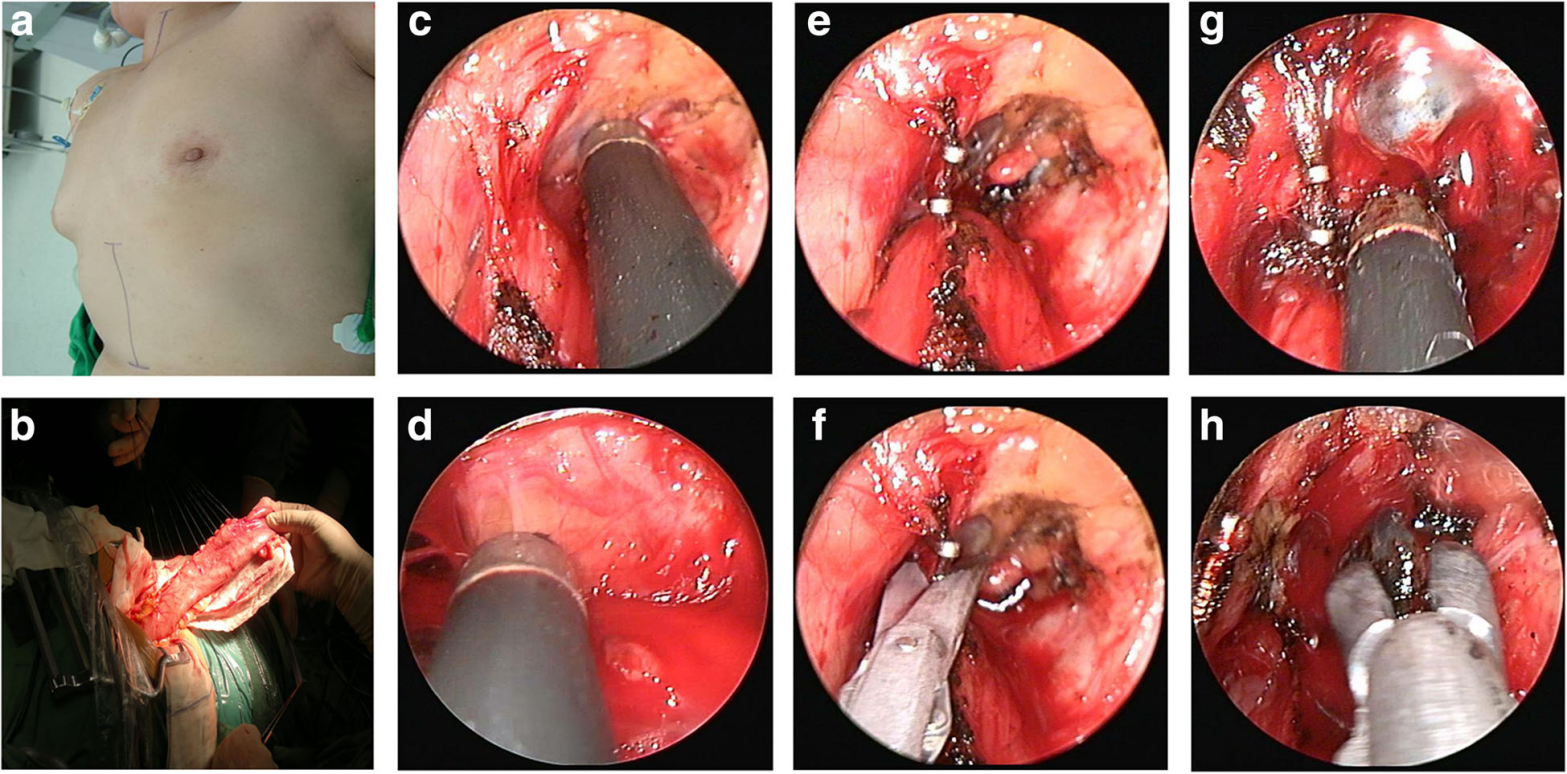
This material was originally published in [Mediastinoscopy-assisted oesophagectomy in T1 oesophageal cancer patients with serious comorbidities: a 5-year long-term follow-up] by / edited by ([12] and Oxford University Press), and has been reproduced by permission of Oxford University Press [http://global.oup.com/academic]. **a** The patient positioning and surgical approach. **b** Gastric mobilization was performed via epigastria midline incision. **c**, **d** The coagulation/aspiration was used to dissect the thoracic esophagus along the esophageal bed. **e**, **f** The esophageal artery was handled by titanium clips under mediastinoscopy. **g** and **h** show the resection of mediastinal lymph nodes under mediastinoscopy

The MAE procedure was performed as previously described [12]. Patients were in the horizontal position. The cervical esophagus was dissected via the incision of anterior border of sternomastoid muscle (Fig. 1a). The electric coagulation/aspiration was used to dissect the thoracic esophagus along the esophagus bed (Fig. 1c, d). The titanium clips were used for dealing with esophageal artery (Fig. 1e, f). The mediastinal lymph nodes were resected through the use of coagulation/aspiration and biopsy forceps (Fig. 1g, h). The epigastria midline incision was adopted to dissect the stomach and perform the abdominal lymphadenectomy (Fig. 1b). Gastroesophagostomy was performed in the neck by using the hand-sewn method. The nasal feeding tube was placed.

The Ivor Lewis procedure was performed as described below. Patients were in the supine position to dissect the stomach and perform the abdominal lymphadenectomy via midline incision on the upper abdomen. Patients were changed to the left lateral position after abdominal surgery. A posterolateral incision in the fifth intercostal space was used to dissect thoracic esophagus and clear away the thoracic lymph nodes. Esophagogastric anastomosis was performed in the uppermost part of the thorax. Patients were changed to the supine position again to place the nasal feeding tube. The Ivor Lewis procedure and MAE were performed by the same team.

### Propensity score matching

Propensity score matching was adopted to match subjects in the MAE and Ivor Lewis groups. The propensity scores were calculated by gender, age, tumor location, tumor length, histological grading, and nodal status. Using the nearest neighbor method, caliper value 0.01, and 1:1 matching algorithm, 19 of 31 patients undergoing MAE and 19 of 81 patients undergoing the Ivor Lewis procedure were matched and analyzed.

### Pathological examination

Pathological examination was performed to assess the tumor morphology, resection margin, histological grading, vessel invasion, tumor length, lymphatic metastasis, infiltration depth, and lymphatic metastasis. The eighth edition of the TNM classification for esophageal carcinoma maintained by the American Joint Committee on Cancer (AJCC) was adopted for the postoperative pathological staging.

### Statistical analysis

We followed up these patients until October 2016 via rediagnosis, telephone calls, and letters. Data were expressed as mean ± standard deviation. The main endpoint of this study was the OS. A statistical software package SPSS 22.0 (SPSS, Inc. IL, USA) was used. Continuous variables were compared using *T* tests. Categorical variables were compared using the chi-square test. Fisher’ exact test was adopted if necessary. The OS was analyzed by the Kaplan–Meier method and Cox multivariate regression analysis. Statistical significance was defined as *P* < 0.05.

## Results

### Patient characteristics and laboratory findings

Thirty-eight T2 esophageal carcinoma patients who underwent MAE (*n* = 19) and the Ivor Lewis (*n* = 19) procedure in our department were included in this nested retrospective study. The preoperative clinical data of these patients are given in Table 1. No statistically significant difference was found between the groups in terms of gender, age, tobacco use, forced expiratory volume in one second (FEV1.0), FEV1.0/forced vital capacity (FVC), PaO_2_, creatinine, blood glucose, ejection fraction (EF), or prothrombin time before operation.

**Table 1:** Patient characteristics and laboratory findings of the patients

	MAE	Ivor Lewis	*P*
Male/female	12:7	13:6	0.732
Median age, year	62	62	0.237
Mean age, year	63.1 ± 7.1	63.2 ± 4.8	0.794
Smoker/nonsmoker	7:12	8:11	0.740
FEV1.0, L	2.38 ± 0.48	2.49 ± 0.43	0.465
FEV1.0/FVC (%)	81.77 ± 11.18	84.37 ± 8.28	0.426
PaO_2_, mmHg	84.75 ± 10.10	86.58 ± 9.13	0.587
Creatinine, μmol L^−1^	80.80 ± 12.59	84.18 ± 16.49	0.486
Blood glucose, mmol L^− 1^	5.13 ± 0.88	5.43 ± 1.56	0.466
EF (%)	60.1 ± 3.3	58.8 ± 2.9	0.488
PT, s	11.20 ± 0.84	11.42 ± 0.86	0.436

*FEV1.0* forced expiratory volume in one second, *FVC* forced expiratory volume, *EF* ejection fraction, *PT* prothrombin time

### Perioperative conditions

Postoperative complications were assessed as described before [14]. The conditions during perioperative period of the patients are given in Table 2. No statistically significant difference was found between the groups in terms of postoperative hospital stay, anastomotic fistula, pulmonary infection, laryngeal recurrent nerve injury, arrhythmia, chylothorax, and incision infection. The MAE group showed shorter operation time (143.2 ± 20.6 vs 176.8 ± 31.1 min, *P* = 0.001) and less volume of drainage in 24 h (119.2 ± 235.1 vs 626.3 ± 396.3 mL, *P* < 0.001) compared with the Ivor Lewis group. The retention time of the thoracic tube also significantly reduced in the MAE group (27.8 ± 24.0 h, *P* < 0.001) than in the Ivor Lewis group (101.2 ± 54.6 h). The MAE group also showed less hemorrhage during operation (255.4 ± 159.8 vs 367.4 ± 150.9 mL, *P =* 0.059).

**Table 2:** Perioperative conditions of the patients

	MAE	Ivor Lewis	*P*
Operation time, min	143.2 ± 20.6	176.8 ± 31.1	0.001
Hemorrhage in operation, mL	255.4 ± 159.8	367.4 ± 150.9	0.059
Drainage in 24 h, mL	119.2 ± 235.1	626.3 ± 396.3	< 0.001
Retention time of the thoracic tube, h	27.8 ± 24.0	101.2 ± 54.6	< 0.001
Postoperative hospital stay, days	11.1 ± 7.2	11.3 ± 6.5	0.998
Anastomotic fistula	3	1	0.290
Pulmonary infection	0	1	> 0.99
Laryngeal recurrent nerve damage	1	0	> 0.99
Arrhythmia	2	0	0.486
Gastric retention	0	3	0.230
Chylothorax	1	1	> 0.99
Incision infection	3	1	0.640

### Postoperative pathological findings

The eighth edition of the AJCC classification for esophageal carcinoma was adopted for the postoperative pathological staging. The postoperative pathological findings are listed in Table 3. No significant difference was found in tumor location, tumor morphology, resection margin, histological grading, vessel invasion, tumor length, nodal status, TNM stage, and number of positive lymph nodes. The MAE group showed less dissected lymph nodes (12.2 ± 5.4 vs 16.8 ± 5.8, *P* = 0.044) than the Ivor Lewis group, especially in the upper mediastinum (1.8 ± 2.1 vs 3.5 ± 2.3, *P* < 0.001) and middle mediastinum (2.5 ± 2.0 vs 5.3 ± 3.2, *P* = 0.027).

**Table 3:** Postoperative pathological findings

	MAE	Ivor Lewis	*P*
Tumor location (middle/lower)	13:6	15:4	0.461
Tumor morphology (ulcer/medullary/masses)	13:2:4	13:4:2	0.513
Resection margin (R0:R1)	19:0	18:1	> 0.99
Histological grading (G2:G3)	12:7	10:9	0.511
Vessel invasion	1	2	0.547
Tumor length	2.8 ± 0.7	2.9 ± 0.8	0.665
Nodal status (N0:N1:N2:N3)	13:4:2:0	14:2:2:1	0.636
TNM stage (IIa: IIIa: IIIb:IVa)	13:4:2:0	14:2:2:1	0.636
Number of positive lymph nodes	0.8 ± 1.6	1.0 ± 2.4	0.281
Dissected lymph node number	12.2 ± 5.4	16.8 ± 5.8	0.044
Upper mediastinum	1.8 ± 2.1	3.5 ± 2.3	< 0.001
Middle mediastinum	2.5 ± 2.0	5.3 ± 3.2	0.027
Lower mediastinum	2.1 ± 1.9	1.9 ± 1.4	0.850
Abdominal	5.8 ± 2.9	5.6 ± 3.0	0.687
Postoperative radiotherapy	6	8	0.501
Postoperative chemotherapy	9	8	0.744

### Long-term outcomes

**Fig. 2:**
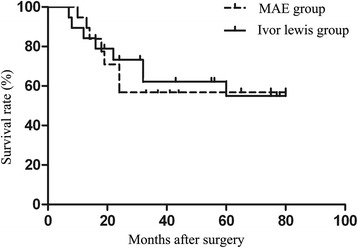
Overall survival curves after MAE and Ivor Lewis procedure. The mean survival time was 59.1 months [95% confidence interval (CI) 45.2–72.9] for the MAE group and 53.3 months (95% CI 38.2–68.4) for the Ivor Lewis group. No significant difference was found in the long-term survival rate between the two groups (*P* = 0.635)

We followed up these patients until death or October 2016. The median follow-up time was 31.0 months (7–80). The mean survival time was 59.1 months [95% confidence interval (CI) 45.2–72.9] for the MAE group and 53.3 months (95% CI 38.2–68.4) for the Ivor Lewis group (Fig. 2). No significant difference was found in the OS between the two groups (*P* = 0.635). Results of the multivariate analysis by Cox regression demonstrated that the nodal stage was an independent prognostic factor for these patients (*P* = 0.016). The surgical method was not an independent prognostic factor for these patients (*P* = 0.290). Postoperative radiotherapy (*P* = 0.595) was not an independent prognostic factor for these patients, as well as the postoperative chemotherapy (*P* = 0.731) (Table 4).

**Table 4:** Results of the Cox regression model for the analysis of prognosis

	*P*	HR	95.0% CI for Exp(B)	
			Lower	Upper
Age	0.454	0.970	0.897	1.050
Tumor location	0.505	0.725	0.281	1.868
Tumor type	0.260	0.749	0.454	1.237
Tumor differentiation	0.354	0.537	0.148	1.951
Vessel invasion	0.592	0.588	0.084	4.012
Nodal stage	0.016	3.155	1.548	5.383
Tumor length	0.379	0.728	0.369	1.477
Postoperative radiotherapy	0.595	1.443	0.373	5.582
Postoperative chemotherapy	0.731	1.189	0.444	3.181
MAE/Ivor Lewis	0.290	0.579	0.377	5.549

## Discussion

ESC occurs with high morbidity and mortality in China [3]. Esophageal resection is regarded as the cornerstone of treatment for esophageal carcinoma patients to date. Transthoracic esophagectomy is a complex procedure and associated with high surgical risk [15]. Thoracotomy can be avoided in MAE. However, MAE is still not widely acceptable because of the controversy about lymphadenectomy. Our previous study showed that MAE for patients with T1 esophageal carcinoma was safe and had a satisfied long-term survival rate [12]. However, more studies are needed for advanced esophageal carcinoma patients. This nested case-control study explored MAE versus Ivor Lewis procedure for T2 middle and lower thoracic esophageal carcinoma patients. The complications and outcomes of Ivor Lewis and MAE groups were retrospectively analyzed.

The MAE group was advantageous over the Ivor Lewis group in terms of shorter operation time. In the MAE procedure, esophagectomy using mediastinoscopy and gastric mobilization via upper abdominal incision could go on simultaneously [16]. This method greatly shortened the operation time. Changing positions were necessary for patients undergoing the Ivor Lewis procedure. And the abdominal and thoracic regions cannot be operated at the same time.

Opening thorax could be avoided in MAE. If the mediastinal pleura could maintain integrity during operation, the thoracic tube was not used in MAE. The thoracic tube is routinely used in the Ivor Lewis procedure. This procedure shortened the retention time of the thoracic tube in the MAE group. Complete mediastinal pleura could also limit bleeding by pressing, thereby greatly reducing the volume of drainage in 24 h. A previous study showed that the lung function after operation could benefit from protection of the integrity of pleura [17]. The MAE group also showed less hemorrhage during operation compared with the Ivor Lewis group, although the result was not statistically significant (255.4 ± 159.8 vs 367.4 ± 150.9 mL, *P =* 0.059). These results showed that the MAE procedure had less surgical trauma compared with the Ivor Lewis procedure.

In the present study, the Ivor Lewis group also showed more patients with gastric retention compared with the MAE group, although no statistically significant difference was found (Table 2). We fixed the intrathoracic stomach between the mediastinal pleura in MAE. The intrathoracic stomach is fixed to the right thorax in the Ivor Lewis procedure. Right intrathoracic stomach may reduce the stenosis of the stomach [18]. And right intrathoracic stomach may also lead to inappropriate angle between the pylorus and the stomach. This physical factor may increase the risk of delayed gastric emptying.

The result showed that the perioperative conditions of MAE for T2 esophageal carcinoma patients were acceptable and similar to or even superior to those of the Ivor Lewis procedure in some areas. MAE could be performed safely for T2 esophageal carcinoma patients.

Lymph node metastasis is an important factor affecting the OS of esophageal carcinoma patients. According to the TNM classification for esophageal carcinoma, 15 lymph nodes in every surgical specimen are required for reliable nodal status. In the present study, more dissected lymph nodes were found in the Ivor Lewis group than in the MAE group. Inadequate lymphadenectomy was found in the MAE group. A potential risk of tumor under-staging might be resulted in the MAE group. In MAE, the operation field in the mediastinum was restricted, and lymphadenectomy for the thorax was difficult [12]. In the Ivor Lewis procedure, good exposure of the thorax was beneficial for lymph node resections. The lymphadenectomy is a major disadvantage of MAE. This shortage could be covered by ameliorating the surgical technique and improving the surgical instruments [11, 19].

It was reported that more number of resected lymph nodes were correlated positively with survival time [20]. However, some studies reported that survival time was not able to benefit from radical lymphadenectomy [21]. The Kaplan–Meier method showed that there was no significant difference in the long-term survival rate between the groups (*P* = 0.635). Cox regression also showed that the surgical method was not an independent prognostic factor for these patients (*P* = 0.290). Our results showed that the long-term survival rate for patients with T2 esophageal carcinoma who received MAE was acceptable. Previous study showed that tumor length influenced the longtime survival rate [22], while the Cox regression showed that the tumor length was not an independent prognostic factor for patients in this study. This may result from the small sample size of this study.

Transthoracic esophagectomy is involved with high surgical risk. Previous study showed that the perioperative mortality of transthoracic esophagectomy was about 2–3% [15]. It was reported that more than one-third patients who underwent transthoracic esophagectomy had perioperative complication [23]. Our result showed that both the long-term survival rate and the perioperative complications of MAE for T2 esophageal cancer patients were acceptable. MAE may provide a surgical option for T2 esophageal cancer patients, especially for those who cannot tolerate transthoracic esophagectomy. This retrospective nested case–control study was performed at a single center. Hence, randomized, controlled, multicenter clinical trials are needed to confirm the findings.

## Conclusion

MAE procedure showed less surgical trauma compared with the Ivor Lewis procedure. The mediastinal lymphadenectomy of patients with T2 esophageal cancer who underwent MAE was inferior to those who underwent Ivor Lewis procedure. The perioperative complications and OS of MAE group were no worse than the Ivor Lewis group.

## Abbreviations

AJCC: American Joint Committee on Cancer
EF: Ejection fractions
ESC: Esophageal squamous cell carcinoma
FEV: Forced expiratory volume
FVC: Forced vital capacity
MAE: Mediastinoscopy-assisted esophagectomy
OS: Overall survival
